# Degree and direction of overlap between social vulnerability and community resilience measurements

**DOI:** 10.1371/journal.pone.0275975

**Published:** 2022-10-20

**Authors:** Sahar Derakhshan, Christopher T. Emrich, Susan L. Cutter

**Affiliations:** 1 Institute of the Environment and Sustainability, University of California, Los Angeles, Los Angeles, California, United States of America; 2 Hazards Vulnerability and Resilience Institute, University of South Carolina, Columbia, South Carolina, United States of America; 3 School of Public Administration, National Center for Integrated Coastal Research, University of Central Florida, Orlando, Florida, United States of America; 4 Department of Geography, Hazards Vulnerability and Resilience Institute, University of South Carolina, Columbia, South Carolina, United States of America; Tulane University School of Public Health and Tropical Medicine, UNITED STATES

## Abstract

An ongoing debate in academic and practitioner communities, centers on the measurement similarities and differences between social vulnerability and community resilience. More specifically, many see social vulnerability and community resilience measurements as conceptually and empirically the same. Only through a critical and comparative assessment can we ascertain the extent to which these measurement schemas empirically relate to one another. This paper uses two well-known indices—the social vulnerability index (SoVI) and the Baseline Resilience Indicators for Communities (BRIC) to address the topic. The paper employs spatio-temporal correlations to test for differences or divergence (negative associations) and similarities or convergence (positive associations), and the degree of overlap. These tests use continental U.S. counties, two timeframes (2010 and 2015), and two case study sub-regions (to identify changes in measurement associations going from national to regional scales given the place-based nature of each index). Geospatial analytics indicate a divergence with little overlap between SoVI and BRIC measurements, based on low negative correlation coefficients (around 30%) for both time periods. There is some spatial variability in measurement overlap, but less than 2% of counties show hot spot clustering of correlations of more than 50% in either year. The strongest overlap and divergence in both years occurs in few counties in California, Arizona, and Maine. The degree of overlap in measurements at the regional scale is greater in the Gulf Region (39%) than in the Southeast Atlantic region (21% in 2010; 28% in 2015) suggesting more homogeneity in Gulf Coast counties based on population and place characteristics. However, in both study areas SoVI and BRIC measurements are negatively associated. Given their inclusion in the National Risk Index, both social vulnerability and resilience metrics are needed to interpret the local community capacities in natural hazards risk planning, as a vulnerable community could be highly resilient or vice versa.

## 1. Introduction

Determining community resilience to extreme events or hazards is an ongoing line of inquiry in multiple fields ranging from engineering to natural sciences to social sciences and beyond. Central to nearly every conversation on the topic is the conceptual role of vulnerability, especially social vulnerability in enhancing or attenuating resilience. Contemporary literature largely assumes that the concepts of hazard/disaster vulnerability and resilience overlap and are the inverse of one another, making the claim that places with high levels of social vulnerability will exhibit lower levels of community resilience [1, 2]. Such claims have not been empirically tested in meaningful ways to ascertain if these conceptual relationships are true for all places and true over time?

There have been relatively few empirical studies explicitly assessing relationships between community resilience and social vulnerability measures and how these manifest themselves spatially and temporally [3]. Whether resilience and vulnerability are complementary or conflicting concepts, an idea initially posed by Miller et al. [4], remains ambiguous, despite recent studies on reconciling measurements of these two concepts [5]. Are earlier claims of statistical independence and negative correlations true? In other words, what is the degree of overlap and direction of association between vulnerability and community resilience measurements?

Two broad questions frame our research. First, are social vulnerability and community resilience measurements essentially the same—simply describing characteristics from inverse perspectives? In this regard, we would anticipate a strong negative correlation between the two and a large degree of overlap in measurement. Second, are statistical relationships between social vulnerability and community resilience consistent over time and across space or is there inter- and intra-regional variability in their relative independence or degree of overlap?

We address these questions through a series of statistical and geospatial analyses of two well-known indices of social vulnerability and community resilience [6] specifically the Social Vulnerability Index (SoVI) and the Baseline Resilience Indicators for Communities (BRIC) [6]. We first examine the continental U.S. (CONUS) counties for two time-periods, 2010 and 2015. To ascertain inter- and intra-regional convergence or divergence in association we assessed regional variability by re-calibrating social vulnerability and community resilience scores for two different coastal study regions: the U.S. Gulf of Mexico coast and southeast Atlantic coast. The study areas are selected due to their rather similar patterns of hazards in the past years (e.g., tropical storms, and flooding).

## 2. Background

Modeling social vulnerability using a range of indicators and approaches is a robust area of research in the hazards and disasters field. Comparisons of different approaches and composite indicators abound, where the multi-dimensional construct of Social Vulnerability Index (SoVI) allows tracing the contributing to vulnerability at each location [7–10]. One current focus of the field is on validation of social vulnerability indicators and how well they explain disaster outcomes [9, 11–18]. Other validation studies of social vulnerability indices have used specific hazards such as flooding [15] and brushfires [19] to measure adverse outcomes such as economic losses. Measuring temporal changes in social vulnerability is another area of interest looking retrospectively in monitoring change in index scores over time in the U.S. [20] or describing decadal changes in social vulnerability and the dynamic nature of the underlying driving factors and their spatial manifestation [21]. Taking a prospective approach, Hardy and Hauer [22] examined selected demographic characteristics of socially vulnerable populations (poverty, gender, race, age, and ethnicity) in 2010 and projected estimates of individuals in 2050 to ascertain the number of socially vulnerable people at future risk from sea-level rise along the Georgia coast.

The development of empirically based resilience indicators is less robust than that of social vulnerability assessments, largely due to the definitional vagueness of the term resilience, or more specifically community resilience [23]. Johnson et al. [5] attribute this “semantic ambiguity” to the nature of the index construction dichotomized as theory driven versus data driven. There is no dominant approach in resilience assessment, which currently ranges from descriptions of community attributes to measurement of community assets and capacities [6, 24–26], largely due to the myriad of disciplines engaged with the concept and methodological approaches. Validation of resilience indices remains limited at this point with a few exceptions [11, 27, 28] rendering most resilience indices, including the Baseline Indicators for Communities (BRIC), to usage as screening or descriptive tools. However, both SoVI and BRIC are the most robust, well-known, oft-cited and used models. A comparison of an initial set of FEMA indicators with BRIC [29], and a current report on the 20 modified FEMA-CRIA variables [30], highlights the comprehensiveness of 49 BRIC indicators and the distinguishing six resilience components in BRIC is instrumental for this study. Furthermore, the National Risk Index [31] also employs both SoVI and BRIC in their methodology, attesting to their prominence in the field and utility for emergency management and planning [32].

Few studies quantitatively address the overlapping nature of resilience and vulnerability measurements. One of the earliest empirical studies [33] examined two components of community resilience—economic development and social capital—and validated their index for Mississippi counties using an index of social vulnerability (SoVI). Sherrieb et al. only found a 14% overlap between their community resilience measure and SoVI, yet a significantly strong negative correlation between the two (r = -0.68). Two other studies [34, 35] also found negative correlations between community resilience and social vulnerability with varying strengths of the correlations, but none as high as Sherrieb et al. As they concluded, “…data support the premise that the three attributes share some common characteristics yet are distinct concepts (2010, p. 241).” Johnson et al. [5] compared six empirically based indices at the variable level to identify redundancies between them and using a factor analytic approach found five factors accounted for 34% of the variation in county measurements of vulnerability and resilience. However, and most significantly, they did not distinguish between vulnerability and resilience variables and thus did not delineate the overlap in variable level measurements and interactions.

## 3. Materials and methods

The SoVI employed in this study uses the current configuration of 29 variables (for county-level analysis) following the established inductive methodology of the algorithm [36]. This includes normalization by z-scores and Principal Component Analysis (PCA) with Varimax rotation for extracting factors with eigenvalues greater than one. After determining the cardinality of the factors (increasing or decreasing vulnerability), the sum of the equally weighted factors produces the final SoVI score [37, 38]. SoVI scores were generated for two different time periods (2010 and 2015) using American Community Survey data five-year estimates (e.g., ACS 2006–2010, 2011–2015) and the same input variables (see sovius.org) calculated for all 3,108 CONUS counties. Given the place-based and comparative nature of SoVI where scores are sensitive to the study region selected, we recomputed SoVI separately for the two time periods for each study region (Gulf Coast and southeastern Atlantic coast) to assure the accurate representation of intra-regional county social vulnerability.

BRIC’s deductive methodology is rooted in the Disaster Recovery of Place (DROP) Model [39], initially formulated for the U.S. Southeast [40], then reformulated and implemented for all CONUS counties [35], and replicated using 2015 data (ACS 2011–2015) [41] to monitor temporal change. The BRIC takes a hierarchical capitals approach to measuring community resilience by identifying input variables representing six different conceptual areas of resilience—each influencing the assets and capacities within communities that enhance resilience: social, economic, institutional, infrastructure, environmental, and community capitals. Forty-nine input variables represent these generalized capitals. Applying min-max scaling to normalize each variable in their respective sub-index (or capital) produces a range in values from 0 (least resilient) to 1 (most resilient). Averaging the values within each sub-index produces the individual resilience capital score and summing the six capital scores creates a county’s overall BRIC score. Again, due to the place-based nature of the BRIC measurement, we recalculated BRIC for the smaller-study regions using the same input variables but normalized them for each study area using the process of min-max normalization and integration of the sub-indexes for the final BRIC score.

Both geospatial analytics and statistical computations and analyses (using ESRI ArcMapS 10.8.1 and SPSS 27.0 software) are used to gauge associations between vulnerability and resilience scores. Geographically Weighted Regression (GWR)—a local form of linear regression—in ArcMap 10.8.1 assesses spatially varying interactions between SoVI, BRIC, and the six BRIC components in each of the study regions. GWR’s resultant local R^2^ are mapped for each of the pairs (i.e., 42 models), as a measure of dependence between vulnerability and resilience scores (R^2^ range between 0.0 and 1.0 and indicates the goodness of fit for the local regression model), in which SoVI is the explanatory variable and BRIC (including individual resilience components) is the dependent variable. This procedure tests how much of the variations in resilience metrics are explained by social vulnerability. Examination of the standard residuals from the regression ensures they are spatially random with no significant clustering. As both SoVI and BRIC are continuous variables, the Pearson correlation statistic provides a measure of the similarity between them and confirmed using a difference of means t-tests. Finally, SoVI and BRIC scores are classified into three groupings (low, medium, high) by standard deviation from the mean and mapped for visual display.

## 4. Results

### 4.1. Comparisons SoVI and BRIC at the national scale

The degree of statistical overlap between SoVI scores and BRIC scores is first tested for each index separately—BRIC (2010 and 2015) and SoVI (2010 and 2015)—by conducting correlation analysis (Table 1). We define correlations between variables with values above +0.7 or less than -0.7 as collinear (or similar). Positive values for variable correlations are convergent, negative values listed as divergent. The correlation between both SoVI 2010 and 2015, and for BRIC 2010 and 2015 are all above 0.9 (*p<0*.*01*). In other words, there is little change between the different time periods in the index scores for social vulnerability and community resilience, respectively. Correlations between SoVI and BRIC scores are significantly negatively associated (coefficients ranging from -0.275 to -0.315, *p<0*.*01*), suggesting less than 31% statistical overlap between overall vulnerability and resilience measures. There was a slight increase in the overlap between SoVI and BRIC from 2010 to 2015.

**Table 1:** Pearson correlation test results between SoVI and BRIC.

Variable Pairs	Pearson’s r
SoVI 2010 and SoVI 2015	0.908**
BRIC 2010 and BRIC 2015	0.911**
SoVI 2010 and BRIC 2010	-0.275**
SoVI 2015 and BRIC 2015	-0.315**

N = 3107

** Correlation is significant at the .01 level

**Fig 1:**
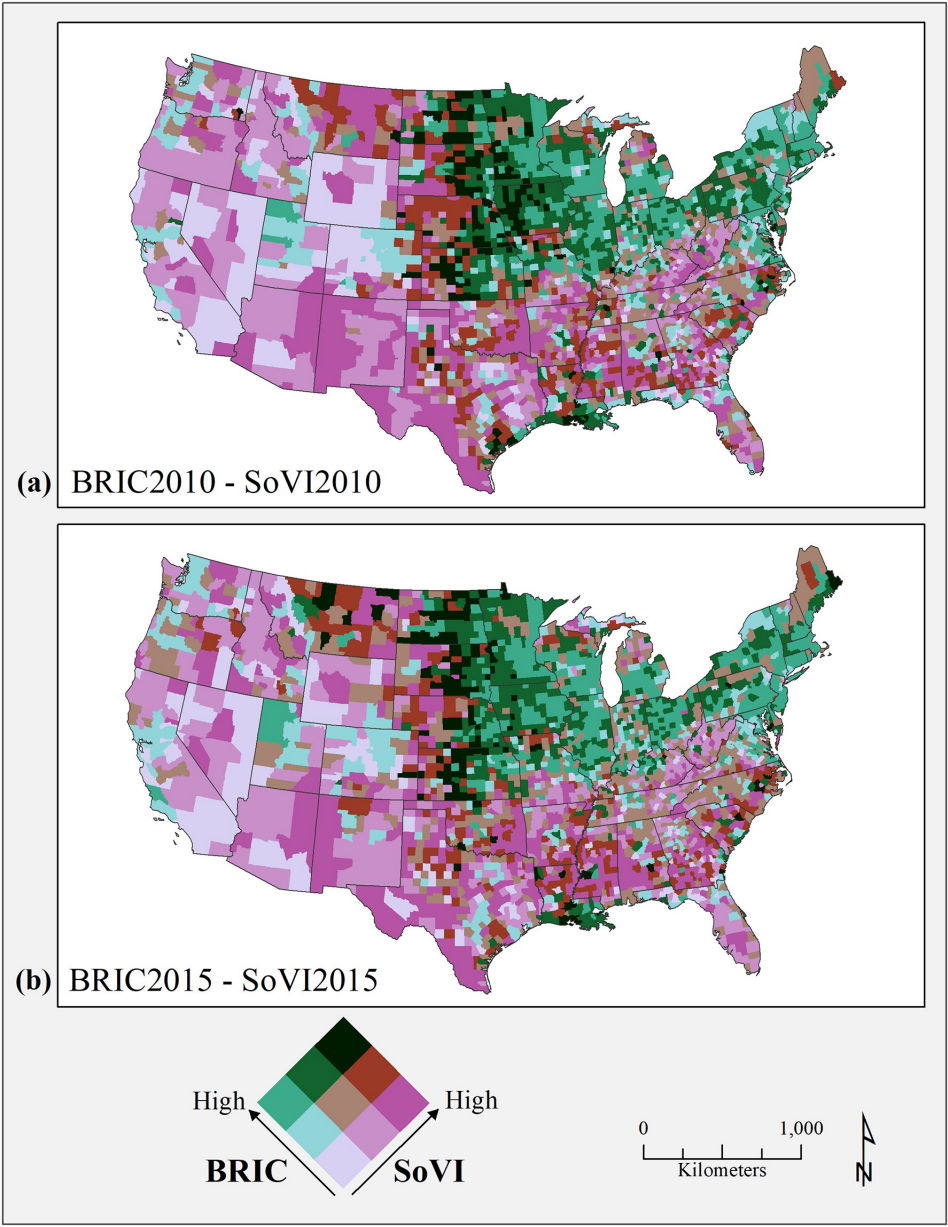
Bivariate maps for BRIC and SoVI (a) 2010 and (b) 2015. County and state boundaries are retrieved from the U.S. Census Bureau (https://www.census.gov/geographies/mapping-files/time-series/geo/carto-boundary-file.html).

The spatial distribution of BRIC and SoVI scores in bivariate maps shows little change in the geographic pattern over the five years (Fig 1). Areas with high resilience and relatively low levels of social vulnerability dominate the northern states stretching from New England through the Great Lakes states and Upper Midwest—the traditional manufacturing core and corn belt region. In contrast, higher levels of social vulnerability with generally low levels of resilience are visible in the southern states, which also contain scattered pockets of counties with moderate to high levels of resilience. The western third of the U.S. shows a mix, but with higher vulnerability than resilience at the county level. Counties with both high levels of resilience and social vulnerability are in the Great Plains states and coastal Louisiana. The higher resilience in these counties is due to relatively more residents born in the state where they now reside, higher English proficiency, more religious organizations, and lower number of mobile homes. Greater social vulnerability reflects lower median house values, higher percentage of employment in extractive industries, age-dependent populations, and social security recipients. For example, both Iberia, Parish in Louisiana and Gregory County in South Dakota are in the high resilience/high vulnerability grouping, but the indicators placing them there are quite different. Iberia Parish in Louisiana scores high on resilience due to higher percentage of native-born residents, wetlands as an environmental flood buffer, and more federal mitigation spending. At the same time, it scores high on social vulnerability because of higher percent of African American female-headed households, lower educational attainment, and less sturdy housing types (e.g., more mobile homes). For Gregory County in South Dakota, disaster aid experience, sturdier housing types, and medical care capacity are the drivers of higher resilience, while employment in extractive industries, higher median age, and age-dependent populations contribute to higher levels of social vulnerability. The counties shaded in dark burgundy color, show the most convergence (i.e., high resilience, high social vulnerability).

**Fig 2:**
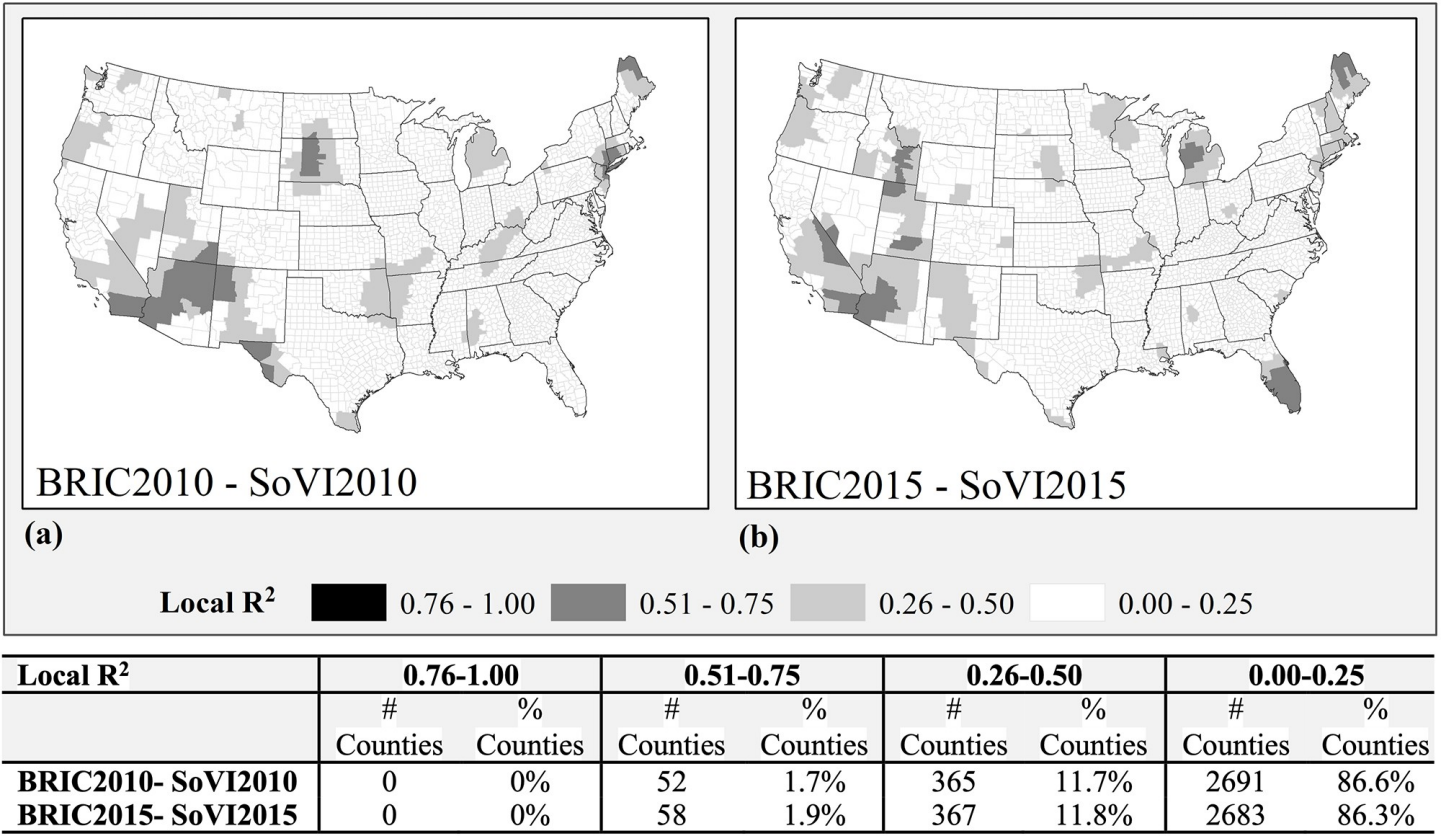
Local R^2^ from GWR results for BRIC and SoVI (a) 2010, (b) 2015. County and state boundaries are retrieved from the U.S. Census Bureau (https://www.census.gov/geographies/mapping-files/time-series/geo/carto-boundary-file.html).

Adjusting for potential spatial autocorrelation of SoVI and BRIC scores, we employed a Geographically Weighted Regression in ArcGIS. Results indicate minimal overlap between BRIC and SoVI in 2010 with even less overlap “between 2010 and 2015 model runs. The degree of consistency has an interesting spatial pattern with most counties (about 86%) showing no significant overlap between BRIC and SoVI measurements (i.e., have less than 0.25 local R^2^ values). However, moderate overlap occurs in New England, the Southwest, southern California, and northern Michigan (Fig 2). There are slightly more areas showing stronger overlaps between SoVI and BRIC in 2015 than in 2010, with south Florida, Great Basin (Utah, Nevada, and eastern Idaho) counties, and central Michigan counties showing the most change. Counties with the strongest correlations between BRIC and SoVI (Local R^2^ >0.5) represent less than 2% of all counties in both years. Further, the strongest temporal overlap between resilience and vulnerability occurs in three counties in southern California (San Diego, Imperial, and Riverside), four in Arizona (Maricopa, La Paz, Yuma, and Yavapai), and one county in Maine (Aroostook). Examination of the distribution of standard residuals for each timeframe does not show any significant clustering.

The spatial and temporal change in correlation between BRIC and SoVI indicate increases in both social vulnerability and resilience in the Northern Rockies and Plains region as well as a few southern counties, while counties with decreasing resilience and social vulnerability are seen scattered in the Upper Midwest and in southern Missouri and Illinois. Counties increasing in resilience while decreasing their social vulnerability appear throughout the nation, as do counties with increasing social and decreasing resilience (n = 29). Overall, only 4% (n = 136) of U.S. counties changed to the highest or lowest category for either BRIC or SoVI, and there is no discernable pattern reflected in the change. The spatial patterns of association for all resilience components in relation to social vulnerability are similar in the two timeframes of 2010 and 2015, but simply constrict in their overall spatial extent.

### 4.2. regional variability of indicator measurements

**Fig 3:**
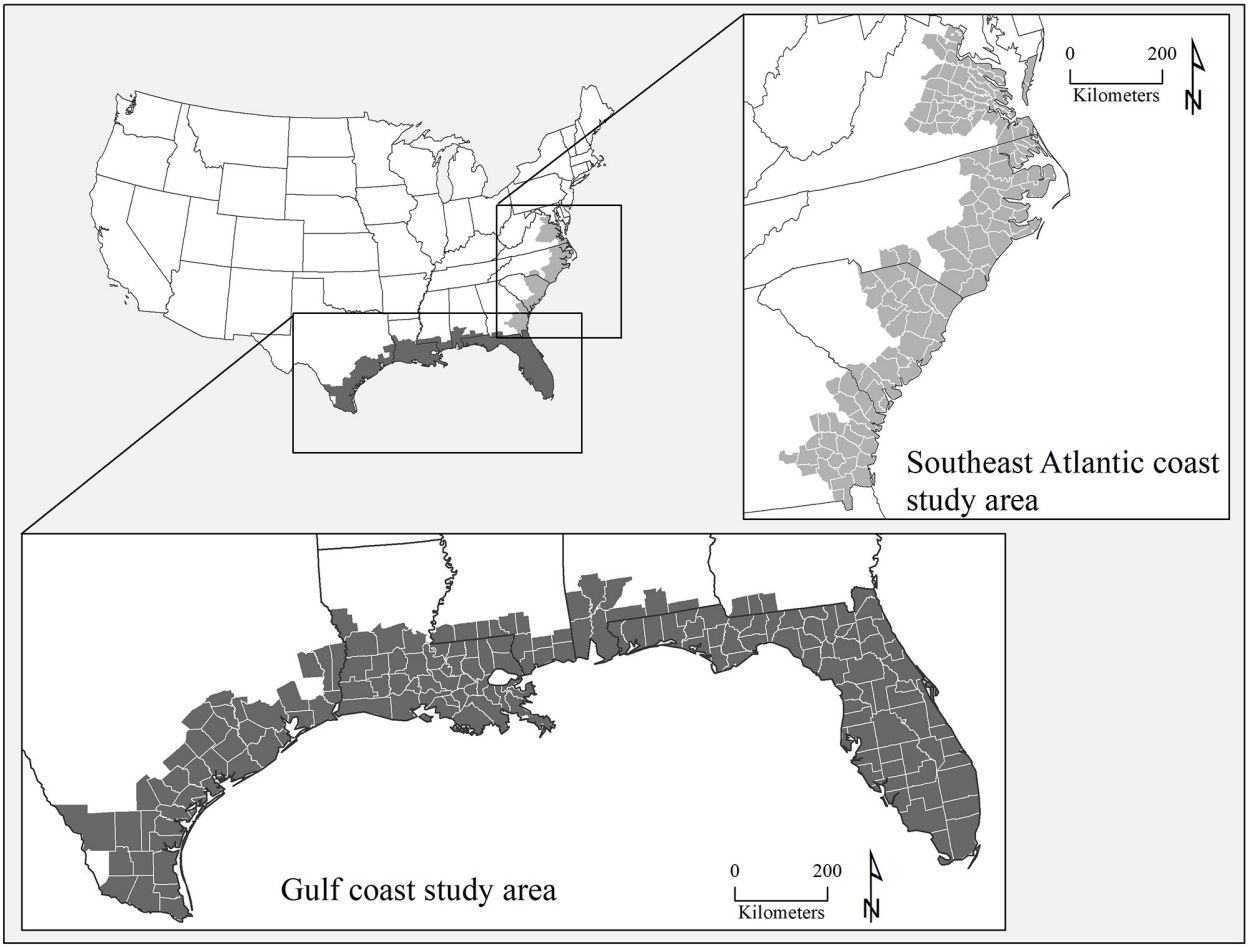
Study regions of Gulf Coast, and southeast Atlantic coast. County and state boundaries are retrieved from the U.S. Census Bureau (https://www.census.gov/geographies/mapping-files/time-series/geo/carto-boundary-file.html).

Given the geographically determinate nature of both the SoVI and BRIC metrics, we would expect to see more homogeneity in social vulnerability, hazard exposure and experience, and resilience at local to regional scales than at the national level. To test this hypothesis, two different coastal study areas (Gulf Coast and southeast Atlantic coast, Fig 3) with similar patterns of hazards in the past years—frequent flooding from heavy rainfall events, tropical storms, and hurricane landfalls—were selected. However, Gulf Coast counties experienced more industrial accidents and environmental contamination from oil and gas spills [42].

Based on NOAA’s Office for Coastal Management [43] definition of coastal counties, the Gulf of Mexico region covers from Texas to Florida and includes 170 counties. The second region includes 146 southeast Atlantic coastal counties from Georgia to Virginia (Fig 3). Because NOAA’s definition of coastal counties covers all of Florida, we included all Florida counties in the Gulf Coast sample to keep Florida intact. However, in the case of Georgia, three southern counties (Thomas, Grady, and Decatur) are included in the Gulf Coast region and other coastal counties are in the southeast Atlantic coast region, since the delineation of these counties already follows the split between these two regions.

All SoVI and BRIC indicators are recalculated (i.e., new z-scores for SoVI and new min-max values for all the 49 BRIC indicators) prior to running the analysis for each regional testbed. For the Gulf Coast, the correlation between the two different SoVI years is high (Pearson’s r = 0.91, *p <* .*01*), as is the correlation for the two BRIC years (Pearson’s r = 0.86, *p <* .*01)*. In the southeast Atlantic coastal region, the correlation between SoVI 2010 and SoVI 2015 is roughly the same as for Gulf Coast counties (Pearson’s r = 0.89, *p <* .*01*), while the correlation between BRIC 2010 and BRIC 2015 is less (Pearson’s r = 0.79, *p <* .*01*). Comparison of the regional downscaling suggests that social vulnerability measurements are stable over the five-year period, while community resilience indicators showed less correlation over time.

The relationship between BRIC and SoVI does change slightly over time between the regions (Table 2). For example, in Gulf Coast counties, there was a 39% overlap between SoVI and BRIC measurements for both years—well above the national average. For southeast Atlantic counties, there was less overlap between BRIC and SoVI measurements in 2010 (21%), but the overlap increased in 2015 to 28%, which was below the national average.

**Table 2:** Pearson correlation test results between SoVI, BRIC, and BRIC components (Gulf coast and southeast Atlantic coast regions).

Variable Pairs	Gulf Coast (Pearson’s R)	Southeast Atlantic Coast (Pearson’s R)
SoVI 2010 and BRIC 2010	-0.389**	-0.210*
SoVI 2015 and BRIC 2015	-0.39**	-0.283**
Number of counties	170	146

** Correlation is significant at the 0.01 level

* Correlation is significant at the 0.05 level

**Fig 4:**
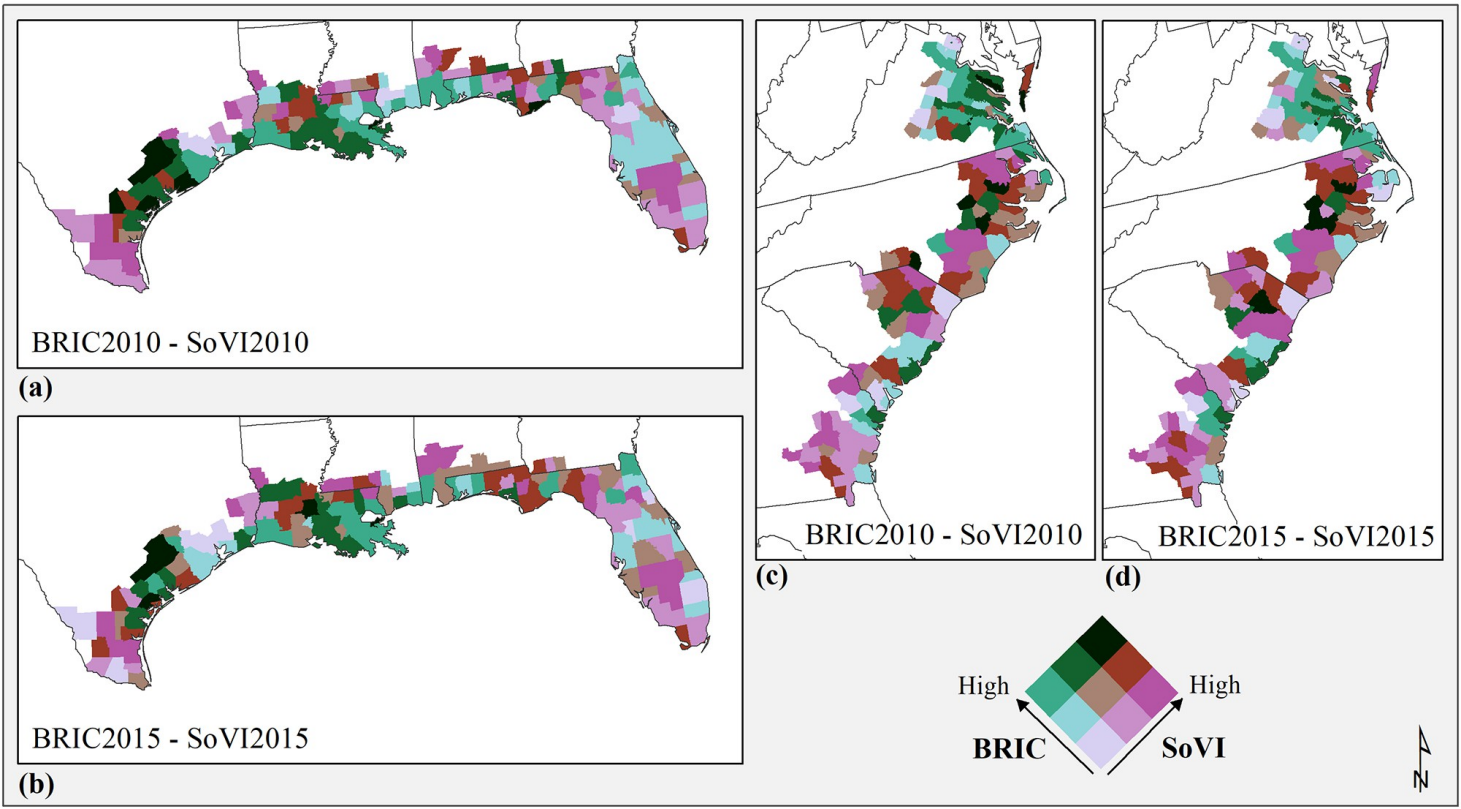
Bivariate maps for BRIC and SoVI Gulf region counties (a) 2010, (b) 2015, and southeast Atlantic coastal counties (c) 2010, (d) 2015. County and state boundaries are retrieved from the U.S. Census Bureau (https://www.census.gov/geographies/mapping-files/time-series/geo/carto-boundary-file.html).

Representing BRIC and SoVI using bivariate classes shows similar temporal patterns from 2010 to 2015 (Fig 4) no clear indication of correlation can be seen. However, the associations are spatially diverse, for example, in the Gulf Coast region (Fig 4a and 4b) the counties of Lavaca, DeWitt, Colorado, Fayette, and Refugio in Texas show areas with high resilience and higher social vulnerability in both timeframes, but the more southern counties of Texas (e.g., Kenedy, Brooks, and Duval), or central counties in Florida (e.g., Okeechobee, Highlands, Hardee, DeSoto, Glades, and Hendry) display lower resilience and high vulnerability. Also, the spatial distribution of classes in the southeast Atlantic region (Fig 4c and 4d) indicates negative associations between BRIC and SoVI, where counties in Virginia have higher resilience and lower vulnerability, while the counties in Georgia have lower resilience and higher vulnerability with two exceptions: Chatham (Savannah) and Bryan (home to Ft. Stewart) counties that have high resilience and low social vulnerability.

**Fig 5:**
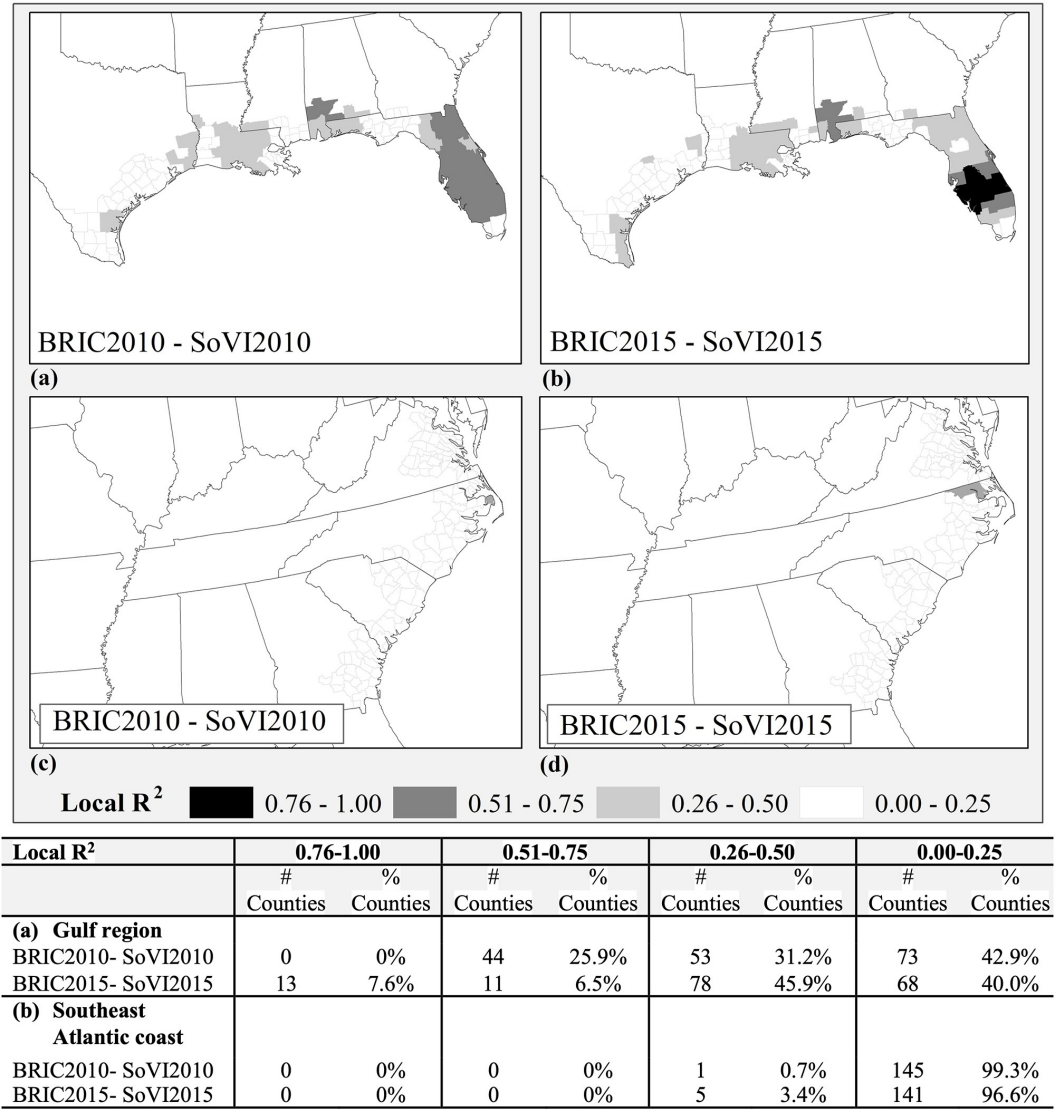
Local R^2^ from GWR results for BRIC and SoVI (2010, 2015)—(a, b) Gulf region counties, (c, d) Southeast Atlantic coastal counties. County and state boundaries are retrieved from the U.S. Census Bureau (https://www.census.gov/geographies/mapping-files/time-series/geo/carto-boundary-file.html).

In the Gulf region, although most counties do not display overlap between SoVI and BRIC there is considerably more similarity in the distribution of local R^2^ for all timeframes with some counties showing increasing overlap from 2010–2015 in the range of R^2^ from 0.25 to 0.5. South Florida is the only area that shows significant overlap (Local R^2^ >0.51) between BRIC and SoVI for both years (Fig 5a and 5b) and an increase in overlap (Local R^2^>0.76 in 2015, especially in central Florida. Testing the distribution of standard residuals for the two timeframes does not show a significant clustering. However, an examination of class changes for BRIC and SoVI shows an increase in social vulnerability in central Florida counties with no change in resilience, which might have caused a locally stronger correlation (i.e., higher Local R^2^) between the two measures here in 2015 (Fig 5b). Similarly, the sequence of no-change in local R^2^ from 2010 to 2015 in the southeastern Atlantic region (Fig 5c and 5d) can be due to a scattered pattern of BRIC and SoVI class changes, which have not altered the overall weak association between the two indicators in this study region.

### 4.3. Explaining temporal change

The driving factors in the spatio-temporal patterns of association between SoVI and BRIC are potentially from changes in population or the input variables. However, differences between results from the two timeframes are not due to population changes based on the lack of significance the clusters and outliers of percent population change (tested with Moran’s I), which show a different spatial pattern (Appendix A in S1 File).

The change in SoVI and BRIC spatial association patterns from 2010 to 2015 in U.S. counties appears to be mostly a result of the change in social vulnerability input variables. We expected to find strong correlations between the individual SoVI variables in 2010 and 2015 (e.g., Pearson’s r > 0.90, p < .001) thus demonstrating their lack of change over the five years. Instead, we found several notable discrepancies in correlations (Appendix B in S1 File) with the largest differences in hospitals per capita, percent speaking English as second language (less than “well”), percent female participation in labor force, percent employment in service industry, people per housing unit, percent children living in 2-parent family, and percent population without health insurance. An examination of the means for these variables in 2010 and 2015 shows significant differences for 86% of CONUS counties (Table 3) for seven of the 29 SoVI input variables.

**Table 3:** Difference of means for SoVI input variables 2010 and 2015*.

Variable	Year	CONUS			Gulf			Southeast Atlantic		
		(3108 counties)			(170 counties)			(146 counties)		
		Mean	t-value	Sig. (2-tailed)	Mean	t-value	Sig. (2-tailed)	Mean	t-value	Sig. (2-tailed)
People per Unit	2010	2.5	41.39	0.000	2.6	9.88	0.000	2.5	8.11	0.000
	2015	2.2			2.3			2.2		
Linguistic Isolation	2010	0.20	5.44	0.000	0.21	1.04	0.299	0.26	2.35	0.019
	2015	0.18			0.19			0.23		
%Female in labor force	2010	46.8	-0.54	0.587	46.2	-0.63	0.527	48.3	-1.14	0.253
	2015	46.9			46.4			48.7		
%Employment in service	2010	17.5	-7.87	0.000	19.2	-2.12	0.035	17.6	-2.77	0.006
	2015	18.3			20.2			18.7		
%Children in 2-parent family	2010	71.5	7.79	0.000	66.3	2.37	0.018	65.0	1.63	0.104
	2015	69.5			64.2			62.9		
Hospitals per capita	2010	0.0	14.46	0.000	0.0	2.96	0.003	0.0	1.77	0.078
	2015	0.0			0.0			0.0		
%Without health insurance	2010	18.3	35.74	0.000	23.8	11.89	0.000	18.5	7.23	0.000
	2015	13.3			17.7			14.7		

*Assumes unequal variances. Includes variables with correlations <0.8 between the two time-periods.

The variation in national patterns between SoVI and BRIC from 2010 to 2015 could be due to changes in household composition (fewer children living in two-parent households), decreases in linguistic proficiency in speaking English, and a significant reduction in the percentage of people without health insurance. It could also be due to changes in the number of community surveys used to create the census value itself—the sample size. Similar tests for the Gulf and southeast Atlantic region counties (Appendix B in S1 File), follow the same change pattern. The variables with correlations below 0.8 are the same as the CONUS (Table 3) and 28% of them have significant difference of means. In the Gulf region 27% of them have significant difference of means. There are a few exceptions like a lower correlation for percent of housing units without access to an automobile in the Gulf region; or a higher correlation for percent of children living in 2-parent families and percent uninsured populations only in the southeast Atlantic region.

Since SoVI and BRIC are constructed differently (PCA vs. Hierarchical), the change in these aspects of communities does not alter the scores of BRIC as much (Appendix B in S1 File). Comparing BRIC variables from 2010 to 2015 shows a major change in input data for telephone access, internet access, psychosocial support facilities, population stability, recent immigrants, disaster aid experience, and civic organizations. Test of difference in means for the BRIC variables that have a correlation below 0.80 shows significant changes for nearly 40% of the input variables in this period (Table 4). These changes mainly demonstrate infrastructure improvements (e.g., telephone and internet access); however, they also support the SoVI variable change explanation that suggests a migratory impact on observed variations in SoVI and BRIC (e.g., population stability and recent immigrants in BRIC, and English proficiency as second language in SoVI).

**Table 4:** Difference of means for BRIC input variables 2010 and 2015*.

Variable	Year	CONUS			Gulf			Southeast Atlantic		
		(3108 counties)			(170 counties)			(146 counties)		
		Mean	t-value	Sig. (2-tailed)	Mean	t-value	Sig. (2-tailed)	Mean	t-value	Sig. (2-tailed)
Educational equality	2010	0.83	14.98	0.000	0.72	4.06	0.000	0.79	3.29	0.001
	2015	0.77			0.63			0.73		
Communication capacity	2010	0.88	-71.84	0.000	0.84	1.71	0.089	0.83	-0.32	0.752
	2015	1.00			0.81			0.83		
Health coverage	2010	0.61	-12.52	0.000	0.65	-1.40	0.161	0.54	2.79	0.006
	2015	0.65			0.68			0.48		
Mental health support	2010	0.04	-25.42	0.000	0.22	4.42	0.000	0.04	-4.68	0.000
	2015	0.08			0.15			0.10		
Health access	2010	0.24	-10.06	0.000	0.47	-0.48	0.633	0.42	1.24	0.216
	2015	0.27			0.48			0.40		
Income equality (Race)	2010	0.49	-73.01	0.000	0.56	1.42	0.156	0.61	2.23	0.026
	2015	0.67			0.53			0.56		
Income equality (Gender)	2010	0.71	-6.28	0.000	0.46	-3.16	0.002	0.69	-2.09	0.038
	2015	0.73			0.53			0.73		
Multi-purpose retail	2010	0.02	-97.20	0.000	0.07	-32.64	0.000	0.11	-8.77	0.000
	2015	0.14			0.52			0.30		
Internet access	2010	0.75	11.86	0.000	0.81	4.04	0.000	0.83	3.30	0.001
	2015	0.67			0.68			0.73		
Sheltering needs	2010	0.05	-5.40	0.000	0.25	3.40	0.001	0.11	-1.73	0.084
	2015	0.06			0.19			0.14		
Place attachment	2010	0.97	23.54	0.000	0.93	6.21	0.000	0.92	4.08	0.000
	2015	0.92			0.82			0.85		
Civic involvement	2010	0.07	28.61	0.000	0.10	5.84	0.000	0.24	9.26	0.000
	2015	0.02			0.03			0.04		
Disaster volunteerism	2010	0.05	-17.86	0.000	0.13	-2.76	0.006	0.14	-1.21	0.225
	2015	0.09			0.18			0.17		
Disaster preparedness	2010	0.04	12.21	0.000	0.06	1.03	0.306	0.06	-6.59	0.000
	2015	0.02			0.05			0.18		
Mitigation spending	2010	0.01	-2.71	0.007	0.08	-4.10	0.000	0.03	0.61	0.543
	2015	0.02			0.20			0.03		
Disaster aid experience	2010	0.13	-10.96	0.000	0.17	-0.26	0.797	0.33	8.15	0.000
	2015	0.18			0.17			0.14		
Population stability	2010	0.92	-27.34	0.000	0.85	1.32	0.187	0.76	-1.86	0.064
	2015	0.97			0.82			0.80		
Local food suppliers	2010	0.02	-5.90	0.000	0.08	-0.97	0.333	0.08	0.57	0.567
	2015	0.03			0.10			0.07		
Efficient energy use	2010	0.90	37.06	0.000	0.57	-0.35	0.725	0.58	0.75	0.454
	2015	0.83			0.59			0.56		

* Assumes unequal variances. Includes variables with correlations <0.8 between the two time-periods.

On the other hand, the BRIC variables for 2010 to 2015 across Gulf and southeast Atlantic counties nearly match the U.S. counties’ results (Appendix B in S1 File), with a few variations. For example, there are weaker correlations for disaster aid experience in the Gulf and in southeast Atlantic counties, or civic organizations in these counties than from the national example. The difference of means test shows significant differences for only half of the variables that have low correlations (Table 4).

## 5. Discussion and conclusion

Quantitative measures of social vulnerability, including the social vulnerability index (SoVI) have been in use since the early 1990s. More recently, empirical measures of community disaster resilience have gained prominence in the United States. A growing debate among social scientists about the similarities and differences between social vulnerability and community resilience necessitated the current work. For many, resilience and vulnerability are seen as “two sides of the same coin” where vulnerable places have low resilience and highly resilient places have low social vulnerability. In response to Johnson et al. [5], this paper advances data-driven approaches for illuminating the relationships between vulnerability and resilience indicators and place-based changes. Our analysis examined geospatial and statistical relationships between two leading indices—social vulnerability (SoVI) and community resilience (BRIC)—at two time-intervals (2010 and 2015), and for a paired subnational comparison of two study regions in the contiguous United States. Our goal was to empirically understand how time, space, and regionality might influence the relationship between indices in terms of their statistical association (positive or negative correlations), and the degree of overlap. In other words, are resilience and social vulnerability measurements merely two sides of the same coin?

*For most the CONUS*, *there is relatively little overlap between SoVI and BRIC measurements*. While negatively correlated, the degree of overlap in measurements ranges from 28% in 2010 to 32% in 2015. In fact, 86% of counties show remarkable congruence for both study years, with a local R^2^ values from 0 to 0.25, or very low correlations between social vulnerability and resilience metrics, spatially and temporally.

*From a regional perspective*, *SoVI and BRIC measurements are generally divergent (negatively correlated)*, *but place-matters in the degree of overlap between the measures*. Although each measure is constructed based on theoretically-informed and nationally consistent data characterizing local/community attributes, the extent of statistical and spatial overlap between vulnerability and resilience shows considerable variability at regional scales. Statistically, county resilience and social vulnerability scores are negatively associated, with similarity in measurement of less than 33% between them for the time-periods when examined for the CONUS Based on the bivariate mapping, there is more convergence with concentrations of high resilience and high social vulnerability counties located in the Great Plains, and low resilience and low social vulnerability counties located in the west especially in southern California and Arizona, and portions of Colorado, Wyoming, and western Nevada.

In the specific analyses for Gulf Coast counties, the degree of overlap between social vulnerability and community resilience measures was similar (39%) for both time-periods suggesting rather homogenous characteristics of both the people and places in the Gulf coast region in terms of social vulnerability and community resilience. In contrast, the southeast Atlantic coastal counties showed a similar negative association between BRIC and SoVI, but the strength was much less, with the degree of measurement overlap growing from 2010 to 2015 (21% to 29%).

*Underlying input data values for both SoVI and BRIC indicator sets between the two time- periods are partially responsible for statistical relationships*. Significant changes in the census collection methods or calculations for both SoVI and BRIC indicators occurring between the two time periods assessed are reflective of either demographic shifts in the populations themselves or results of increased/changing coverage by the US Census data products. For example, percentage increases in immigrant populations coupled with changes in family structure, and more residents speaking English as a second language may have influenced the changes. Furthermore, although counties tend to have lower sample error when compared to other geographies, it must be noted that such error may also influence results. Similarly, changes in internet access and increases in the number of psychosocial support facilities in BRIC for the social resilience may have helped increase the degree of overlap between the two measures. Yet, the alignment was not consistent across CONUS or within our regional studies.

*Across the United States and within the two regional study areas SoVI and BRIC do not explain each other either spatially or statistically*. Taken comprehensively, these results indicate that although few communities might exhibit more alignment between certain aspects of community resilience and social vulnerability, most areas manifest completely opposite representations. While some degree of overlap between the measurements exists as expected, these results are spatially and temporally variant indicating that while SoVI and BRIC negatively correlated, they are not the true opposites or the inverse of one another, given the relative lack of strength in overlap. These findings reflect the significant need for continued research, validations, and knowledge growth in understanding the place-based and comparative contexts explaining how community resilience and social vulnerability measurements diverge or converge as a function of the uniqueness of place.

## Supporting information

S1 File (DOCX)
