# Supplementary material for: Degree and direction of overlap between social vulnerability and community resilience measurements
Source: PLoS One. 2022 Oct 20;17(10):e0275975. doi: 10.1371/journal.pone.0275975 (PMC9584515; doi:10.1371/journal.pone.0275975)
Supplement: S1 File — (DOCX) [file pone.0275975.s001.docx]

# Appendix A


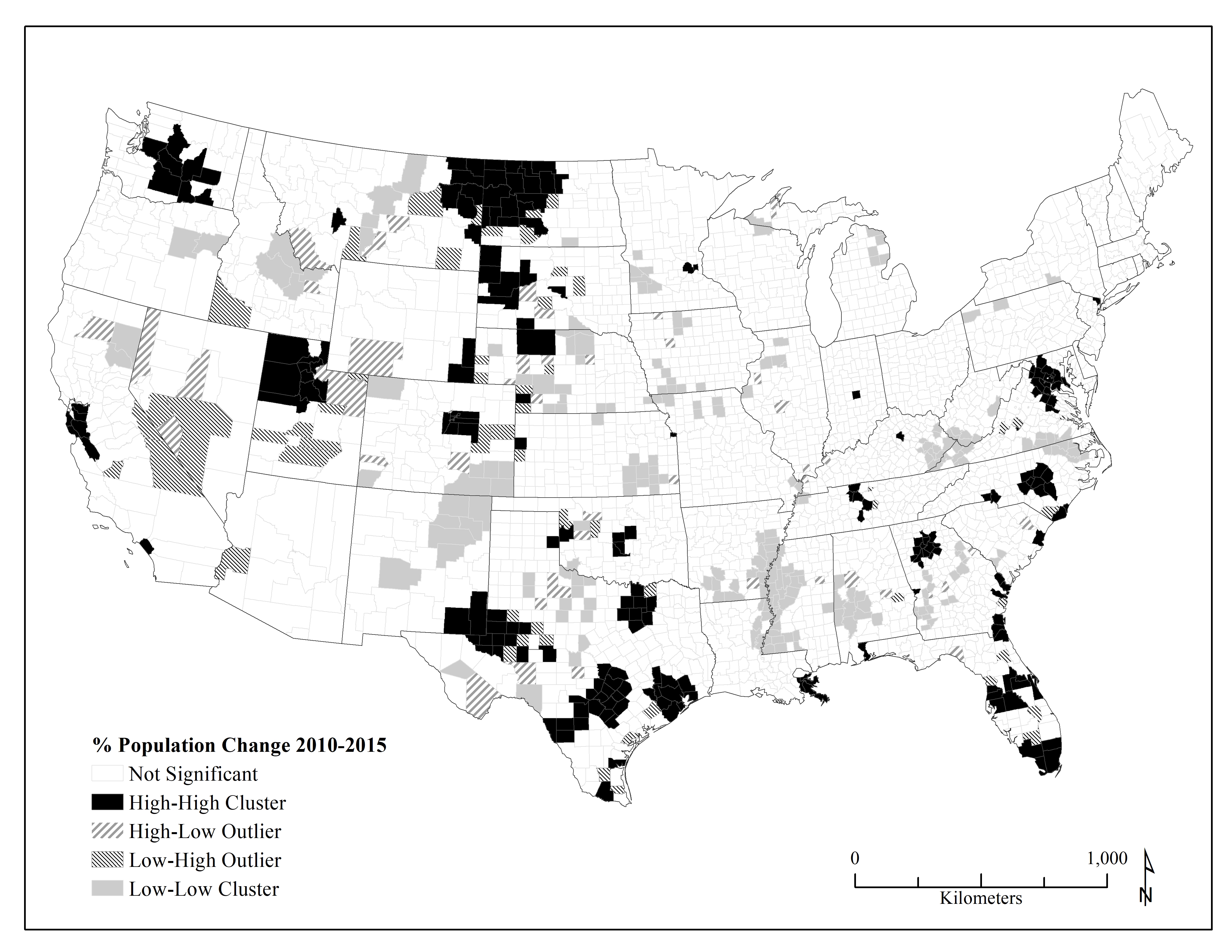


Moran’s I clusters and outliers for percent population change from 2010 to 2015. County and state boundaries are retrieved from the U.S. Census Bureau (https://www.census.gov/geographies/mapping-files/time-series/geo/carto-boundary-file.html)

# Appendix B

The table below presents the correlations (Pearson’s r) between social vulnerability score SoVI and its variables; and community resilience score BRIC and its variables, between 2010 and 2015 (all correlations are significant at *p<0.001*, non-significant correlations are marked as N/A).

|  | **CONUS**  **(3108 counties)** | **Gulf**  **(170 counties)** | **Southeast Atlantic**  **(146 counties)** |
| --- | --- | --- | --- |
| **Variable** | 2010-2015 | 2010-2015 | 2010-2015 |
| **SoVI** | 0.91 | 0.91 | 0.89 |
| Median age | 0.98 | 0.97 | 0.99 |
| % African American | 1 | 1 | 1 |
| % Native American | 0.99 | 0.97 | 0.99 |
| % Asian | 0.99 | 0.99 | 0.99 |
| % Hispanic | 1 | 1 | 0.98 |
| % Age dependent | 0.95 | 0.99 | 0.99 |
| People per unit | 0.73 | 0.78 | 0.71 |
| % Renters | 0.9 | 0.9 | 0.95 |
| % Nursing home residents | 1 | 1 | 1 |
| % Female | 0.92 | 0.98 | 0.94 |
| % Female headed household | 0.94 | 0.9 | 0.92 |
| Per Capita income | 0.92 | 0.94 | 0.98 |
| Linguistic isolation | 0.54 | 0.68 | 0.57 |
| % Unemployment | 0.92 | 0.9 | 0.87 |
| % Poverty | 0.88 | 0.89 | 0.91 |
| % Mobile homes | 0.96 | 0.97 | 0.98 |
| % With less that 12^th^ grade education | 0.94 | 0.94 | 0.92 |
| % Female in labor force | 0.6 | 0.67 | 0.47 |
| % Employed in extractive industries | 0.94 | 0.94 | 0.86 |
| % Employed in service industry | 0.65 | 0.65 | 0.69 |
| % Households receiving social security | 0.89 | 0.94 | 0.94 |
| % Housing units with no car | 0.88 | 0.77 | 0.86 |
| % Children living in 2-parent families | 0.75 | 0.73 | 0.81 |
| % Households earning over $200,000 | 0.89 | 0.9 | 0.97 |
| Median gross rent | 0.94 | 0.91 | 0.96 |
| Median housing value | 0.96 | 0.91 | 0.97 |
| Hospitals per capita | 0.45 | 0.72 | N/A |
| % Population without health insurance | 0.79 | 0.72 | 0.82 |
| % Unoccupied housing units | 0.96 | 0.9 | 0.96 |
| **BRIC** | 0.9 | 0.86 | 0.79 |
| SOCIAL | 0.65 | 0.81 | 0.78 |
| ECONOMIC | 0.86 | 0.56 | 0.80 |
| INFRASTRUCTURE | 0.81 | 0.91 | 0.87 |
| COMMUNITY CAPITAL | 0.86 | 0.86 | 0.74 |
| INSTITUTIONAL | 0.80 | 0.82 | 0.83 |
| ENVIRONMENTAL | 0.80 | 0.85 | 0.86 |
| Educational equality | 0.79 | 0.83 | 0.88 |
| Age | 0.97 | 1 | 0.99 |
| Transportation access | 0.92 | 0.84 | 0.87 |
| Communication capacity | 0.12 | 0.58 | 0.84 |
| Language competency | 0.97 | 0.97 | 0.97 |
| Health insurance | 0.74 | 0.65 | 0.72 |
| Mental health support | 0.15 | N/A | N/A |
| Non-special needs | 0.81 | 0.79 | 0.82 |
| Health access | 0.71 | 0.84 | 0.67 |
| Food access | 0.88 | 0.73 | 0.95 |
| Homeownership | 0.95 | 0.93 | 0.96 |
| Employment | 0.94 | 0.95 | 0.9 |
| Income equality (Race) | 0.72 | 0.73 | 0.87 |
| Primary and tourism employment | 0.92 | 0.89 | 0.78 |
| Income equality (Gender) | 0.75 | 0.83 | 0.78 |
| Business size | 0.89 | 0.86 | 0.9 |
| Federal employment | 0.87 | 0.84 | 0.91 |
| Multi-purpose retail | 0.13 | N/A | 0.19 |
| Housing type (not mobile homes) | 0.97 | 0.98 | 0.98 |
| Medical capacity | 1 | 0.98 | 1 |
| Housing age | 0.92 | 0.83 | 0.9 |
| Industrial re-supply | 1 | 1 | 1 |
| Internet access | 0.45 | 0.59 | 0.44 |
| Temporary housing availability | 0.73 | 0.74 | 0.84 |
| Access/evacuation potential | 0.96 | 0.95 | 1 |
| Sheltering needs | 0.91 | 0.89 | 0.98 |
| Recovery (Public schools) | 0.96 | 0.84 | 0.87 |
| Place attachment (few recent immigrant) | 0.56 | 0.63 | 0.47 |
| Place attachment (nativity/tenure) | 0.98 | 0.99 | 0.99 |
| Political engagement | 0.87 | 0.8 | 0.85 |
| Social capital – Religion | 1 | 1 | 1 |
| Social capital – civic involvement | 0.37 | 0.18 | 0.21 |
| Social capital – Disaster volunteerism | 0.67 | 0.66 | 0.88 |
| Disaster preparedness and response skills | 0.36 | 0.18 | 0.3 |
| Mitigation spending | 0.68 | 0.6 | 0.4 |
| Jurisdictional uniformity | 0.99 | 0.89 | 0.99 |
| Disaster aid experience | 0.49 | 0.33 | 0.24 |
| Flood insurance coverage | 0.92 | 0.97 | 0.83 |
| Local disaster training | 1 | 1 | 1 |
| Population stability | 0.48 | 0.63 | 0.5 |
| Crop insurance coverage | 0.99 | 0.98 | 0.99 |
| Performance regimes- state capital | 0.99 | 1 | 0.98 |
| Performance regimes- nearest metro area | 1 | 1 | 1 |
| Nuclear accident planning | 0.99 | 1 | 1 |
| Local food suppliers | 0.37 | 0.65 | 0.28 |
| Natural flood buffers | 0.97 | 0.97 | 0.98 |
| Efficient energy use | 0.52 | 0.85 | 0.41 |
| Pervious surfaces | 1 | 0.99 | 1 |
| Efficient water use | 0.88 | 0.98 | 1 |
